# Sexual health inequalities among women aged 16-24

**DOI:** 10.1093/eurpub/ckac129.158

**Published:** 2022-10-25

**Authors:** D Solomon, J Gibbs, F Burns, H Mohammed, SJ Migchelsen, CA Sabin

**Affiliations:** 1 Institute for Global Health, University College London, London, UK; 2 NIHR Health Protection Research Unit, University College London, London, UK; 3 Royal Free London NHS Foundation Trust, London, UK; 4 UK Health Security Agency, London, UK

## Abstract

**Introduction:**

Gonorrhoea is the second most commonly diagnosed sexually transmitted infection in England, and diagnoses among young women increased 31% between 2018 and 2019. Understanding the patterns of testing and diagnosis among young women is likely to aid prevention among the most vulnerable segments of this population.

**Methods:**

Data on gonorrhoea diagnoses at sexual health services among women aged 16-24 in England were obtained using the GUMCAD STI Surveillance System. We investigated the relationship between two exposure variables (deprivation and ethnicity), and two outcome variables (number of gonorrhoea tests and number of gonorrhoea diagnoses). Poisson regression was used to calculate rate ratios for the relationship between the exposure and outcome variables. The testing analysis was offset for the size of the population, and the diagnosis analysis was offset for the number of tests within the population.

**Results:**

Between 2012 and 2019, gonorrhoea testing and diagnosis rates were highest among women living in the most deprived areas. The rate of testing in the least deprived 10% of neighbourhoods was significantly lower than that seen in the most deprived 10% of neighbourhoods (rate ratio (RR) 0.79; 95% confidence interval 0.79 - 0.80), and the rate of diagnosis in the least deprived 10% of neighbourhoods was around a third of that seen in the most deprived 10% of neighbourhoods (0.35; 0.33 - 0.36). When compared to White British women, the rate of gonorrhoea diagnosis was lower among Bangladeshi (RR 0.89; 0.75 - 1.05), Indian (0.76; 0.68 - 0.84), Pakistani (0.87; 0.77 - 1.00) and Chinese women (0.60; 0.51 - 0.71) and was highest among Black Caribbean (2.26; 2.18 - 2.33) and Black African (1.40; 1.34 - 1.45) women.

**Conclusions:**

This analysis found inequalities in the distribution of gonorrhoea among young women in England that may indicate structural barriers to STI prevention that are affecting Black women and those living within the most deprived populations.

**Key messages:**

• Gonorrhoea testing rates among young women in England are highest among women from deprived areas and Black women.

• Gonorrhoea diagnosis rates among young women in England are highest among women from deprived areas and Black women.

